# A case report of disseminated nocardiosis with ocular involvement in a myasthenia gravis patient and literature review

**DOI:** 10.1186/s12883-019-1482-4

**Published:** 2019-10-21

**Authors:** Shuhui Wang, Bin Jiang, Yao Li, Yanchang Shang, Zhengshan Liu, Yongbo Zhang

**Affiliations:** 1 Department of Neurology, Beijing Friendship Hospital, Capital Medical University, Beijing, 100050 China; 2 Department of Geriatric Neurology, Chinese People’s Liberation Army General Hospital, National Clinical Research Center for Geriatric Diseases, Beijing, 100853 China; 3 Department of Neurology and Center for Translational Neuromedicine, University of Rochester Medical Center, New York, NY USA

**Keywords:** Disseminated nocardiosis, Myasthenia gravis, Opportunistic infection, Case report

## Abstract

**Background:**

Nocardiosis is a rare and life-threatening opportunistic infection in immunocompromised patients. Myasthenia gravis (MG) patients are potentially at risk of nocardia infection because of the use of immunosuppressive agents. To date, only 7 patients with MG have been reported to have nocardiosis. Disseminated nocardiosis with ocular involvement has not been reported in MG patients.

**Case presentation:**

A 66-year-old man with MG who was receiving treatment with methylprednisolone and azathioprine was found to have a respiratory infection. He also had heterogeneous symptoms with skin, brain and ocular manifestations. Nocardia bacteria verified by the culture of puncture fluid, and a diagnosis of disseminated nocardiosis was made. Except for left eye blindness, the patient completely recovered from the disease with combination antibiotic therapy. To further understand nocardiosis in patients with MG, we reviewed the previous relevant literature. According to the literature, this is the first report of disseminated nocardiosis with ocular involvement in an MG patient.

**Conclusions:**

MG patients with immunosuppressant treatments are potentially at risk of a rare nocardia infection, and a favourable prognosis can be achieved through early diagnosis and appropriate antibiotic therapy.

## Background

Nocardiosis is an acute purulent or chronic granulomatous disease caused by infection of nocardia bacteria, a gram-positive branching rod-shaped aerobic bacterium from the genus Actinomyces [1, 2]. Nocardia is an opportunistic pathogen with a relatively low incidence, and it is usually infectious in immunocompromised patients, such as those with autoimmune disease or those receiving immunosuppressive treatments. They can cause local or disseminated nocardiosis in humans. Patients with myasthenia gravis (MG) are commonly treated with immunosuppressive agents for a long time and are potentially at risk of nocardia infection.

Nocardiosis is a life-threatening infectious disease, especially in patients who cannot be treated appropriately in the early stage of the disease. However, the heterogeneous clinical presentations of affected patients and laboratory test limitations make the diagnosis of nocardiosis very difficult. Therefore, it is important for physicians to diagnose the disease and treat the patients in a timely manner. Here, we present a case of disseminated nocardiosis in an MG patient in whom multiple systems were involved. Based on our findings, we also conducted a literature review.

## Case presentation

A 66-year-old Chinese man presented with fever, cough, dyspnoea and lumbodynia after falling from exercise equipment on July 6, 2015. He had a medical history of blood hypertension and diabetes mellitus. In 2014, he was hospitalized because of fluctuating ptosis and dysphagia. The fatigue test and neostigmine test were positive. Slow frequency repetitive nerve stimulation (3 Hz) on the bilateral facial nerves showed that the compound muscle action potential (CAMP) decrement was more than 15%. An immunological serum test showed that the patient was negative for the acetylcholine receptor (AChR) and muscle-specific kinase (MusK) antibodies but positive for Titin and ryanodine receptor (RyR) antibodies. Thoracic enhanced computed tomography (CT) did not find an abnormal thymus. The patient was diagnosed with generalized MG and received glucocorticoids and pyridostigmine therapy. The maximum dose of methylprednisolone was 56 mg per day, which was subsequently tapered to 20 mg per day. In addition, he took 100 mg azathioprine daily for MG therapy.

A physical examination revealed that the patient had a temperature of 38 degrees centigrade. Other vital signs were in the normal range. There were multiple rales in the bilateral lungs on auscultation. Multiple, irregular, and tender masses were found on the patient’s chest, back, neck, and right limbs. These masses had a high temperature and were red in colour. There was an infectious ulcer mass on the right lower limb. Except for weakness in the right lower limb (5−/5), there were no other abnormal findings on a neurological examination.

Routine laboratory investigations, including routine blood tests, hepatic and renal functions, electrolyte and coagulation function, were in the normal range. Antinuclear antibody (ANA), extractable nuclear antibody (ENA), and neoplastic marker tests were in the normal range. Immunoglobulin (Ig) and complement (C) levels were checked twice. The patient’s levels of IgG/IgA/C3/C4 were normal, but his levels of IgM were 25 mg/dL and 25.7 mg/dL (normal limit, 40–230 mg/dL). The proportions of lymphocyte subgroups (CD3, CD4, CD8, CD4/CD8, CD19, and CD16 + CD56) were normal. Respiratory virus screening was negative. His level of C reactive protein (CRP) was 37 mg/L (normal range < 5 mg/L). Tuberculosis infection T lymphocytes and anti-tuberculosis antibody were not positive. Antibodies against hepatitis B virus (HBV), hepatitis C virus (HCV), human immunodeficiency virus (HIV) and syphilis were negative.

**Fig. 1:**
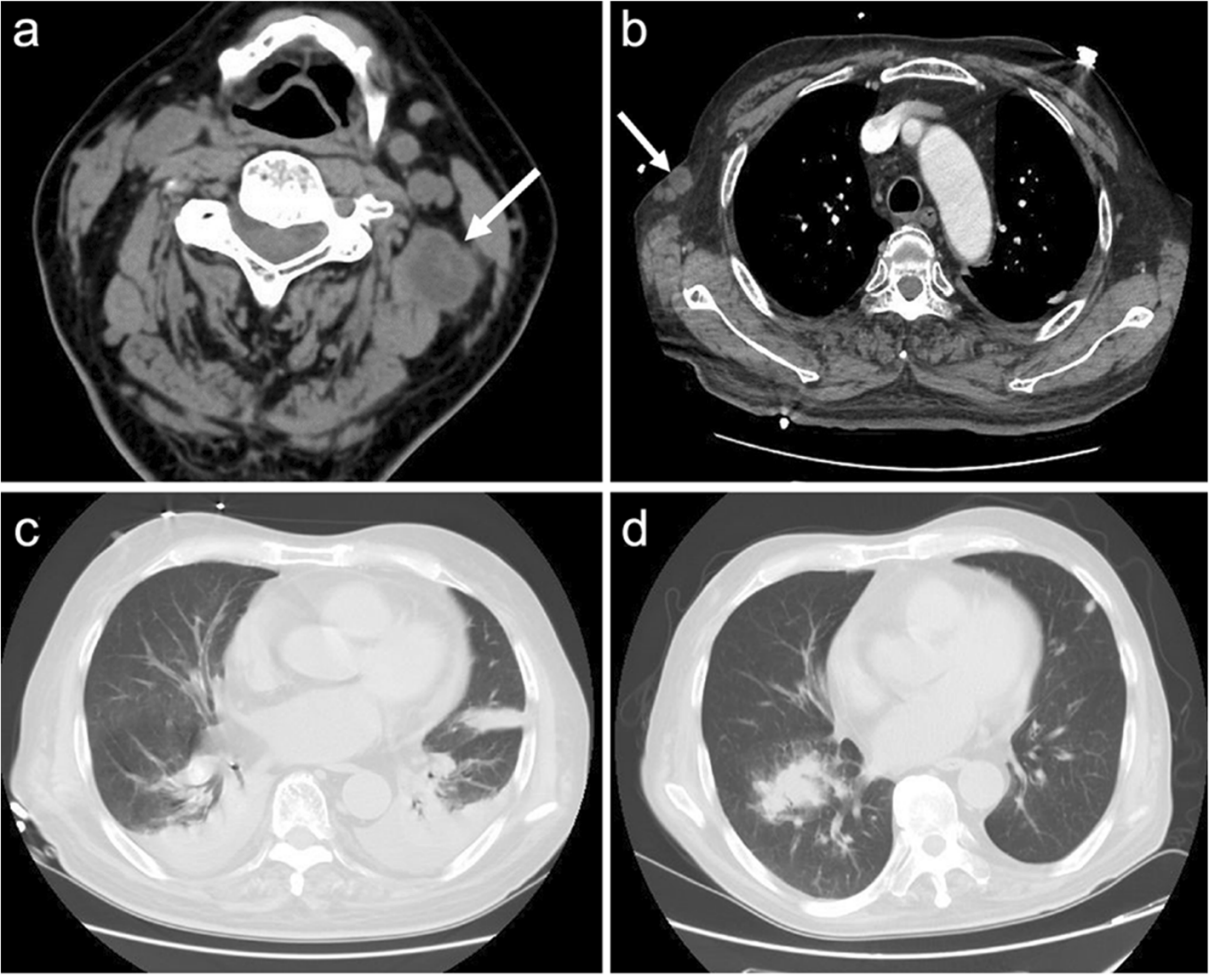
CT images of the neck and chest on the day of admission to hospital (**a, b, c**): a CT scan showing a mass on the left of the neck, arrow shown in (**a**). CT of the chest showing the subcutaneous nodes, with the arrow shown in (**b**). There were multiple nodular or patchy shadows in the bilateral lungs and pleural and pericardial effusion (**c**). One month after treatment with SMZ-TMP, a repeated chest CT (**d**) showed pleural effusion and that the lesions in the left lung had disappeared while the lesions in the right lung had become more apparent

A lumbar spine X-ray scan showed compression fractures in the L1 and L2 vertebral bodies. A neck CT scan found a mass on the left, and a diagnosis of swollen lymph node, abscess or tumour was considered (Fig. 1a). A chest CT scan revealed multiple nodular and patchy shadows in the bilateral lungs and pleural as well as pericardial effusion (Fig. 1b-c). An abdominal CT scan revealed gallstones and a small cyst on the right kidney.

**Fig. 2:**
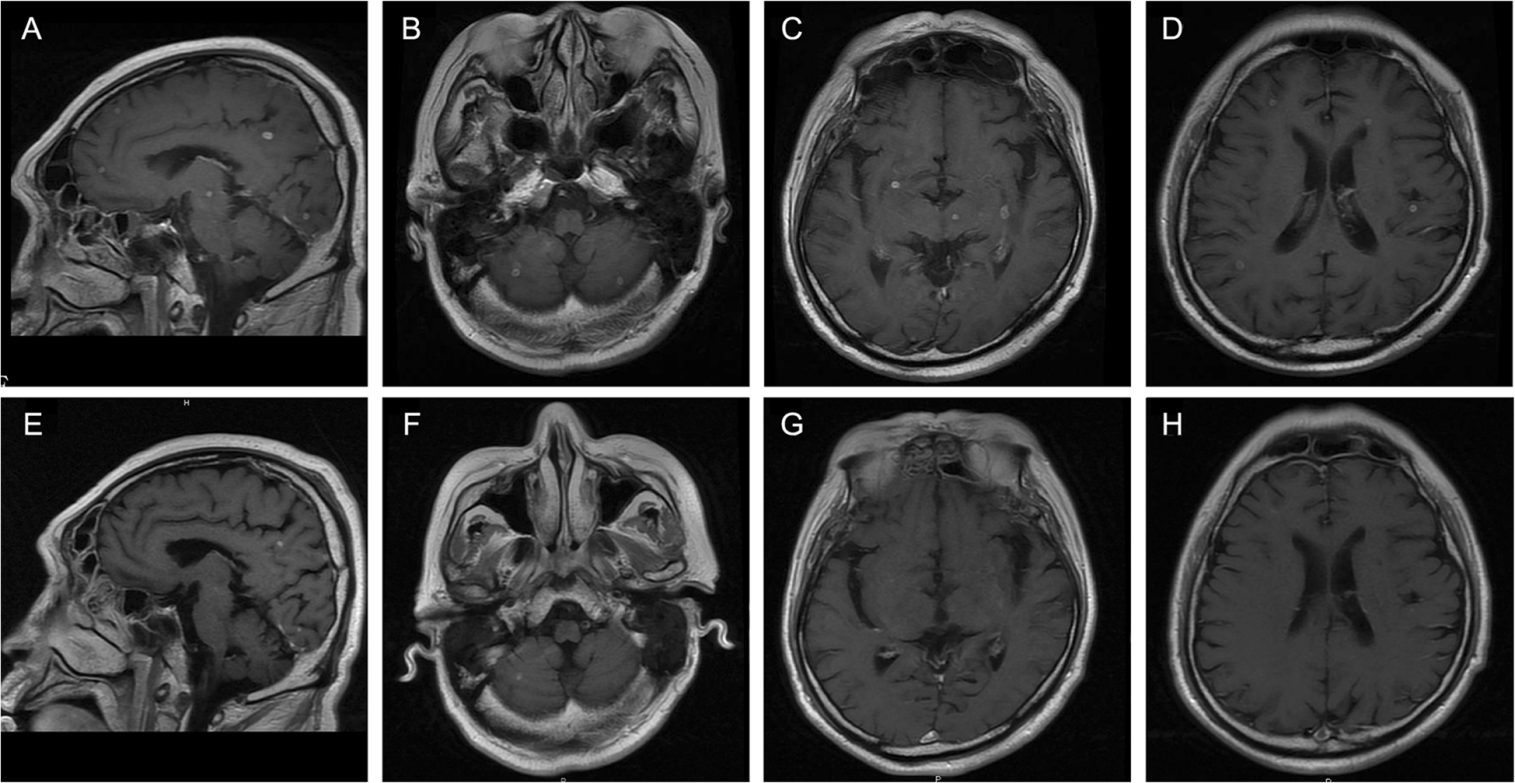
Magnetic resonance imaging of the brain. Gadolinium contrast T1 images, with sagittal reformat (**a**) and axial reformat (**b, c, d**) images of the brain showing ring-enhancing lesions in the bilateral brain and cerebellum. **e-h**: Reviewed MRI scan of the brain showing that some lesions had decreased and some had disappeared after one and half months of SMZ treatment

**Fig. 3:**
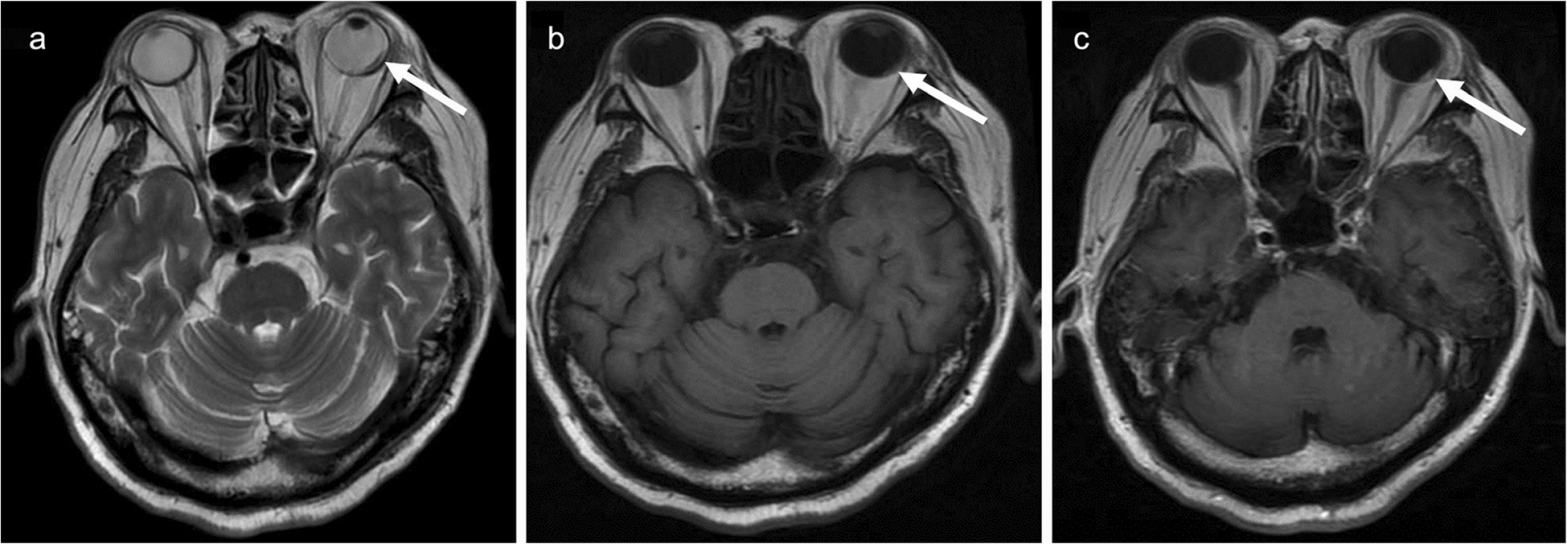
Magnetic resonance imaging of the orbit. An abnormal lesion was noted behind the left eye (shown on arrow) with a high-intensity signal on T2-weighted images (**a**), low signal intensity on T1-weighted images (**b**) and high signal intensity on gadolinium contrast T1-weighted images (**c**)

The patient was diagnosed with pneumonia and received moxifloxacin treatment. The patient’s body temperature gradually returned to the normal range, and cough and dyspnoea were also relieved after treatment. However, he had a fever with chill, heavy dyspnoea, headache, and pain in the right leg after 20 days of treatment. He also felt great pain in the left eye, and his vision became blurred and rapidly aggravated to blindness. For differential diagnoses, both brain CT scan and magnetic resonance imaging (MRI) were performed. The CT scan showed multiple low-intensity lesions in different brain areas, and MRI revealed marked multiple abnormalities in the bilateral cerebrum and cerebellum with low signal intensity on T1-weighted images as well as high signal intensity on T2-weighted images. After administration of gadolinium contrast material, these lesions demonstrated ring enhancement (Fig. 2a-d). Accordingly, a diagnosis of abscess or multiple brain metastases was considered. Importantly, MRI of the orbit showed an abnormal enhanced lesion behind the left eyeball and retinal detachment (Fig. 3a-c). To identify the characteristics of these lesions, we performed percutaneous drainage from the mass on the left neck guided by ultrasound. Nocardia bacteria were found in a culture of puncture fluid, confirming the diagnosis of disseminated nocardiosis.

After the diagnosis of nocardiosis, the patient was treated with trimethoprim-sulfamethoxazole (TMP-SMX) tablets (TMP0.08 g + SMX0.4 g/per tablet, 2 pills every 8 h). Moreover, imipenem/meropenem/ceftriaxone and sporanox were also given for the bacteria and fungus infection. Two weeks later, the patient was found to have leukocytopenia and underwent a bone marrow puncture examination. The results indicated a suspected diagnosis of myelodysplastic syndrome (MDS). Therapy with TMP-SMX tablets was adjusted to 1 pill every 12 h plus injection with etimicin sulfate. Azathioprine therapy was replaced with berbamine to treat the leukocytopenia. Three weeks later, the patient was treated with TMP-SMX tablets alone (1 pill every 12 h). After this treatment, except for the left eye blindness, all of his symptoms, including fever, dyspnoea, headache, and pain in the leg and left eye, gradually remitted. In addition, 1 month of TMP-SMX treatment also reduced pleural effusion and bilateral lung lesions based on repeat chest CT results (Fig. 1d). After one and a half months of TMP-SMX treatment, multiple brain lesions had decreased or even disappeared based on a repeat brain MRI (Fig. 2 e-h). The patient was continuously treated on an out-patient basis with TMP-SMX tablets (1 pill every 12 h) for one and a half years. His blood cells were monitored every 2 weeks. Six months after discharge, the subcutaneous masses had disappeared, and a repeat brain MRI showed that the encephalic lesions had also disappeared. At that point, the dosage of methylprednisolone was tapered slowly until it was stopped because of the improvement observed in his MG, and there were no recurrent syndromes at the 2-year follow-up.

## Discussion and conclusions

In general, nocardia bacteria are opportunistic bacteria that mainly infect immunocompromised patients. Although MG is an autoimmune disease that requires immunosuppressive treatment, nocardia infection is rarely observed in these patients. We performed the literature review by searching the MEDLINE database using the key words “nocardia,” “nocardiosis, “and “myasthenia gravis.” We found that up until our search, only 7 case reports of MG patients with nocardiosis had been reported [3–9]. To improve understanding of the disease process, we summarized the clinical characteristics of these patients, and these are listed in Table 1. Seven out of 8 patients had a good prognosis. All patients were treated with glucocorticoids and other immunosuppressants. Three patients had thymoma, and 1 patient had Good’s syndrome. There was no clear relationship between nocardia infection and MG or thymoma. However, patients with Good’s syndrome, who usually have thymoma and immunodeficiency, easily acquire opportunistic infections. Although our patient had low IgM levels, he had no thymoma and normal levels of lymphocyte subgroups, and a diagnosis of Good’s syndrome was therefore not considered. Repeated immunoglobulin tests showed that he had low levels of IgM and normal levels of IgG, IgA and lymphocyte subgroups. Selective immunoglobulin M deficiency (sIgMD) was considered in our patient and is a rare primary immunodeficiency disease characterized by low levels of IgM (below two standard deviations of the mean) in association with infection and normal levels of IgG and IgA [10]. In addition, our patient had the history of diabetes for many years. Therefore, immunosuppressant use, sIgMD and diabetes were the predisposing factors for nocardia infection in our patient. We concluded the data in Table 1. The following factors may be preconditions for nocardia infection in MG patients. The primary factor was the use of immunomodulator and/or immunosuppressive agents, consistent with numerous previous studies [11, 12]. The second factor was age. We found that MG patients with nocardia infection were 49–79 years old, indicating that older patients with MG might be more susceptible to nocardiosis. This is consistent with the fact that older MG patients have a higher incidence of tumours, such as thymoma, and low immunity. The last factor is gender, as supported by our finding that 7 out of 8 MG patients with nocardia infection were male patients. In summary, older male MG patients being treated with immunomodulator and/or immunosuppressive agents might represent a population that is susceptible to nocardia infection.

**Table 1:** Clinical characteristics of MG patients with disseminated nocardiosis

Case	Age /sex	Author/year	Symptoms	Underlying disease	Immune suppression	Operation	Sites of disease	Treatments	Outcome
1	59/male	Kim 2002	skin lesions, fever, cough	MG, thymoma	prednisolone	thymectomy	skin, lung	TMP-SMX	resolution
2	49/male	El-Herte 2012	Fever, chills, chest pain, weight loss	MG, thymoma	prednisone	thymectomy	lung, brain, heart	imipenem, amikacin, TMP-SMX	resolution
3	59/female	Tanioka 2012	Malaise, cough	MG	prednisolone, tacrolimus, cyclosporine	NA	lung, kidney, brain	imipenem-cilastatin, TMP-SMX, minocycline, meropenem, amikacin, linezolid	resolution
4	79/male	Patel 2013	left leg pain, cough	MG	prednisone	NA	leg, lung	moxifloxacin	NA
5	77/male	Garcia 2015	night sweats, cough	MG, TB	prednisone, plasmapheresis	thymectomy	lung, brain	TMP-SMX, meropenem, linezolid	resolution
6	79/male	Veerappan 2015	leg abscesses	MG	steroid	NA	lung, muscle	vancomycin, piperacillin-tazobactam, ampicillin, ceftriaxone, doxycycline, moxifloxacin, linezolid	resolution
7	52/male	Wargo 2015	cough, dyspnoea, weight loss, subcutaneous nodules, fatigue	MG, thymoma, Good’s syndrome	prednisone, chemotherapy, radiation	NA	lung, skin, brain	meropenem, TMP-SMX, imipenem, linezolid	resolution
8	66/male	Wang 2019	fever, dyspnoea, headache, pain on left eye and right leg, skin ulcer, blindness, muscle strength decreased	MG, hypertension, diabetes	methylprednisolone, azathioprine	None	Lung, brain, skin, ocular	TMP-SMX, etimicin sulfate, moxifloxacin, ceftriaxone, imipenem, meropenem	resolution

Abbreviations: *MG* myasthenia gravis, *TMP-SMX* trimethoprim-sulfamethoxazole, *TB* tuberculosis, *NA* not available

The clinical manifestations of nocardia infections are very heterogeneous and nonspecific. Lung, brain and skin are the most commonly affected sites [13]. All 8 patients had lung lesions. The infection also involved the muscles, heart, and kidneys. In addition to lung, brain and skin lesions, our patient also had ocular lesions. To the best of our knowledge, this is the first case report of an MG patient with disseminated nocardiosis with ocular lesions.

Ocular tissue is an unusual site for disseminated nocardiosis, and ocular infection is generally diagnosed as local nocardiosis, with presents a keratitis or endophthalmitis resulting from ocular trauma or surgery [14]. Occasionally, ocular infection might also be caused by haematogenous dissemination via the choroidal circulation [15]. The ocular nocardia infection that occurred in our patient may have been caused by haematogenous spread because the patient did not have eye trauma or a history of surgery and he had no abnormal signs in his eyeball. The prognosis of ocular nocardiosis is generally poor. Blindness is a common consequence, and ophthalmectomy is performed in approximately 30% of these patients. For these reasons, regular ophthalmologic screening should be performed in patients with suspected disseminated nocardiosis [15, 16]. In our patient, the ocular lesion was located behind the left eyeball, which finally led to retinal detachment. After he received suitable treatment, the ocular lesion completely vanished, but his vision was not restored.

Due to the paucity of trials, there are no formal guidelines to direct drug choice and treatment duration in nocardiosis. Most clinicians agree that CNS nocardiosis warrants a long course of treatment, with 12 months commonly recommended [17]. Empirical treatment of disseminated nocardiosis usually involves three antibiotics, including imipenem or ceftriaxone, TMP-SMX, and amikacin. TMP-SMX is thought to be the cornerstone of treatment for nocardia infections and is also the drug of choice for cerebral nocardiosis due to its good penetration into the CNS. Other drugs, including meropenem, cefotaxime, minocycline, moxifloxacin, levofloxacin, linezolid, tigecycline, and amoxicillin/clavulanic acid, are also used for the treatment of these patients [12]. MG patients should be treated with the proper antibiotics because some antibiotics can aggravate the disease. Patients with nocardiosis often have an underlying autoimmune disease or are receiving immunosuppressive treatment. Therefore, a combination of antibiotics is recommended in the beginning, and a single drug can be maintained after the clinical symptoms are relieved [12]. Immunosuppressive therapy will increase the risk of infection and the difficulty of treating infection in patients with MG. The use of immunosuppressants in MG patients with infections is an important issue. By reviewing the literature [18] and combining our findings with our own clinical practice experience, we cautiously suggest that if the infection can be controlled, immunosuppressive therapy can be continued in MG patients. However, when an infection is hard to control with administration of the proper antibiotics and becomes life-threatening, physicians should reduce the dose of immunosuppressants or even stop it. We stopped the use of azathioprine and continued a tapered dose of methylprednisolone in our patient when he developed leukocytopenia.

Because of the nonspecific manifestations of nocardiosis, most patients with nocardia infection are not diagnosed in the early stage of the disease, and it normally takes from 42 days to 12 months after the appearance of symptoms to achieve a clear diagnosis [19, 20]; this causes a substantial physical and emotional burden in these patients. The prognosis of nocardiosis depends on the location and severity of the infection as well as the overall condition of the patient. The curable rate of pulmonary nocardiosis is approximately 90% with timely treatment, while the one-year mortality rate is high in patients with CNS involvement. The mortality rate is 10-fold higher in patients with solid organ transplantation from nocardiosis than in those without [11, 12]. None of the eight patients included in Table 1 had a history of organ transplantation. Although 4 of the 8 patients had CNS lesions, these lesions were relatively small, the symptoms were mild, and the diagnosis and treatment were timely, and this may have been the reasons for their good prognosis.

In conclusion, nocardiosis is a life-threatening infectious disease, and diagnosis in the early stage and appropriate antibiotic therapy are crucial to prognosis. Ocular involvement is rare in disseminated nocardiosis, and patients who are suspected to have disseminated nocardiosis should receive ophthalmologic screening. Neurological physicians should be aware of nocardia infection in MG patients.

## Data Availability

The datasets generated during and/or analysed during the current study are available from the corresponding author on reasonable request.

## Abbreviations

AChR: Acetylcholine receptor
ANA: Antinuclear antibody
C: Complement
CAMP: Compound muscle action potential
CNS: Central nervous system
CRP: C reactive protein
CT: Computed tomography
ENA: Extractable nuclear antibody
HBV: Hepatitis B virus
HCV: Hepatitis C virus
HIV: Human immunodeficiency virus
Ig: Immunoglobulin
MDS: Myelodysplastic syndrome
MG: Myasthenia gravis
MRI: Magnetic resonance imaging
MusK: Muscle-specific kinase
NA: Not available
RyR: Ryanodine receptor
sIgMD: selective immunoglobulin M deficiency
TB: Tuberculosis
TMP-SMX: Trimethoprim-sulfamethoxazole
